# System Architecture of a European Platform for Health Policy Decision Making: MIDAS

**DOI:** 10.3389/fpubh.2022.838438

**Published:** 2022-03-31

**Authors:** Xi Shi, Gorana Nikolic, Scott Fischaber, Michaela Black, Debbie Rankin, Gorka Epelde, Andoni Beristain, Roberto Alvarez, Monica Arrue, Joao Pita Costa, Marko Grobelnik, Luka Stopar, Juha Pajula, Adil Umer, Peter Poliwoda, Jonathan Wallace, Paul Carlin, Jarmo Pääkkönen, Bart De Moor

**Affiliations:** ^1^Department of Electrical Engineering (ESAT), Stadius Center for Dynamical Systems, Signal Processing and Data Analytics, KU Leuven, Leuven, Belgium; ^2^Vlerick Business School, Leuven, Belgium; ^3^Analytics Engines, Belfast, United Kingdom; ^4^School of Computing, Engineering and Intelligent Systems, Ulster University, Londonderry, United Kingdom; ^5^Vicomtech Foundation, Basque Research and Technology Alliance (BRTA), Donostia-San Sebastián, Spain; ^6^EHealth Group, Biodonostia Health Research Institute, Donostia-San Sebastián, Spain; ^7^Quintelligence, Ljubljana, Slovenia; ^8^AI Lab, Institute Jozef Stefan, Ljubljana, Slovenia; ^9^Data-Driven Solutions, Smart Health, VTT Technical Research Centre of Finland, Tampere, Finland; ^10^IBM Ireland Lab, Innovation Exchange, International Business Machines Corporation, Dublin, Ireland; ^11^School of Computing, Ulster University, Jordanstown, United Kingdom; ^12^Faculty of Wellbeing, Education and Language Studies, Open University, Belfast, United Kingdom; ^13^Centre for Health and Technology, University of Oulu, Oulu, Finland

**Keywords:** public health, decision support system, epidemiology, data visualization, machine learning

## Abstract

**Background:**

Healthcare data is a rich yet underutilized resource due to its disconnected, heterogeneous nature. A means of connecting healthcare data and integrating it with additional open and social data in a secure way can support the monumental challenge policy-makers face in safely accessing all relevant data to assist in managing the health and wellbeing of all. The goal of this study was to develop a novel health data platform within the MIDAS (Meaningful Integration of Data Analytics and Services) project, that harnesses the potential of latent healthcare data in combination with open and social data to support evidence-based health policy decision-making in a privacy-preserving manner.

**Methods:**

The MIDAS platform was developed in an iterative and collaborative way with close involvement of academia, industry, healthcare staff and policy-makers, to solve tasks including data storage, data harmonization, data analytics and visualizations, and open and social data analytics. The platform has been piloted and tested by health departments in four European countries, each focusing on different region-specific health challenges and related data sources.

**Results:**

A novel health data platform solving the needs of Public Health decision-makers was successfully implemented within the four pilot regions connecting heterogeneous healthcare datasets and open datasets and turning large amounts of previously isolated data into actionable information allowing for evidence-based health policy-making and risk stratification through the application and visualization of advanced analytics.

**Conclusions:**

The MIDAS platform delivers a secure, effective and integrated solution to deal with health data, providing support for health policy decision-making, planning of public health activities and the implementation of the Health in All Policies approach. The platform has proven transferable, sustainable and scalable across policies, data and regions.

## Introduction

We live in a data-rich society, which provides extensive opportunities for the development of big data and artificial intelligence technologies to provide new insights to enhance decision-making. Such technologies have particular importance in healthcare and health policy making. Despite the urgent need and opportunity, their use has not reached full potential in this field for various reasons, for example, healthcare data is typically heterogeneous and disconnected, existing in isolated silos, making meaningful analysis difficult. Privacy concerns create an additional barrier in exploiting the potential of healthcare data, preventing data sharing in a timely manner.

A systematic review on big data applications biomedical research and healthcare summarized the big data applications for clinical informatics and public health information (1). Among the studies on clinical informatics applications, most of the platforms were developed for data storage and retrieval (2, 3), data sharing (4, 5), and data security (6), which could not provide simulation, forecast or other analytics. Similarly, when the platform was developed for data analysis (7–9), data storage and data processing lost its priority. There are some platforms using social media to track and monitor public opinions, thereby providing evidence for policy decision making (10, 11). These platforms were mainly for infectious disease surveillance. In general, the platforms mentioned above have a focus on one aspect, such as data storage or processing, data analytics, or social media analysis. However, the whole process is all important to support the health policy decision making. An integrated platform including all these functions is in need.

A means of connecting healthcare data and integrating it with additional open and social data in a secure way did not exist prior to the MIDAS platform release. Such a solution can support the monumental challenge policy-makers face in safely accessing all relevant data to assist in managing the health and wellbeing of all.

The MIDAS project set out to address this challenge and has developed a novel health data platform that connects a range of heterogeneous health-related data with open and social data and applies advanced analytics techniques to provide a visual data-driven decision making tool that enhances healthcare policy making, whilst ensuring key aspects of ethics, security and privacy are adhered to (12). The platform has been piloted across four European regions: Basque Country (Spain), Finland, Northern Ireland (United Kingdom), and Ireland, addressing major health challenges in each region including mental health issues of young adults, diabetes and the aging population, childhood obesity, and social care for children, respectively.

This paper will present the system architecture of the MIDAS platform, which integrates data warehouse, data analytics, data visualization, and external applications for social media analysis, and enables rapid adjustments to new pilots.

## Methods

**Figure 1:**
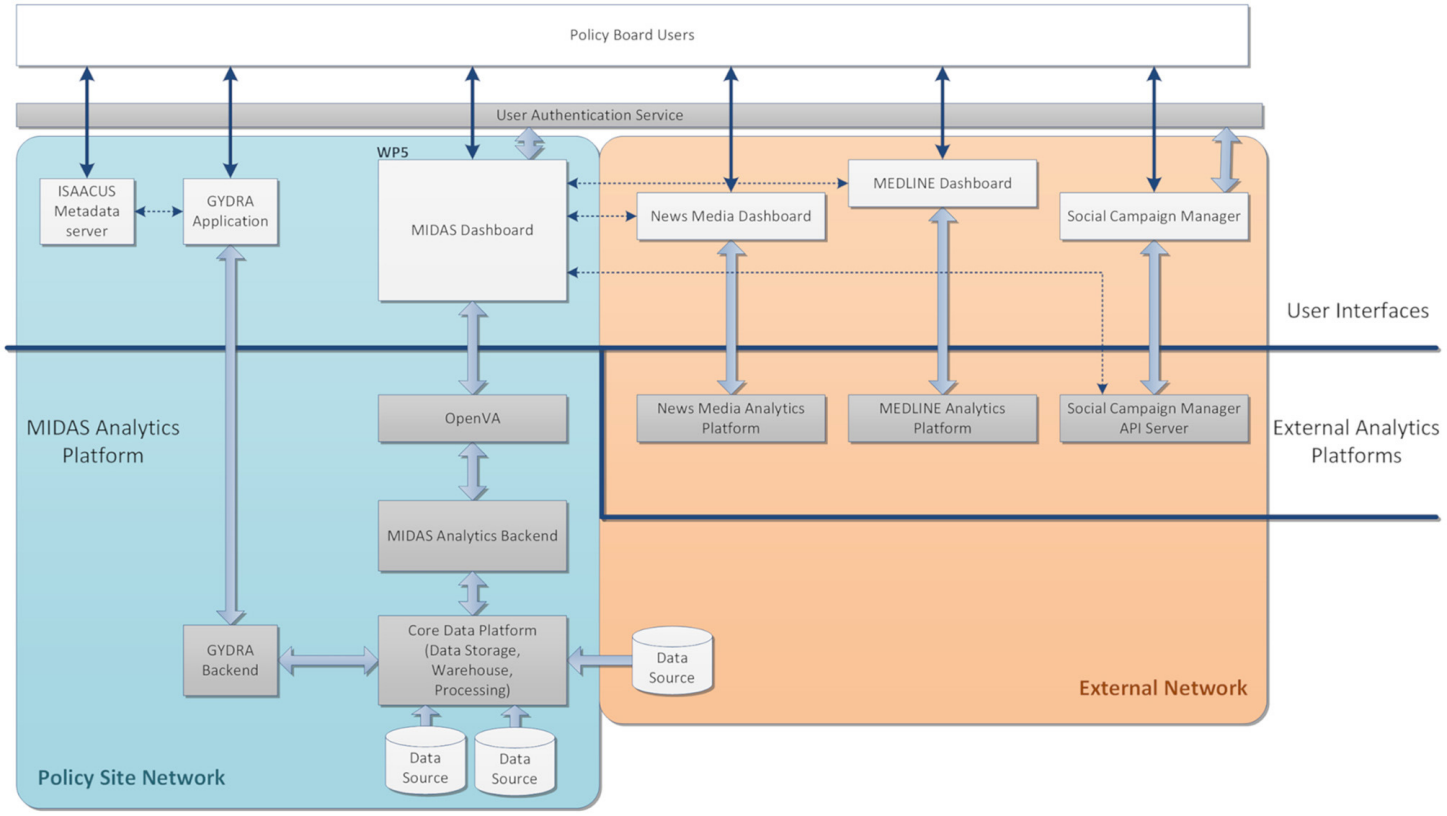
MIDAS Platform Overview. The platform consists of software hosted within a Policy Site Network for analyzing local data and applications hosted externally (External Network) for analyzing open and social data as indicated by the colored boxes. The light-gray boxes indicate end user-facing web-applications, connected to back-end applications and analytics platforms shown in the dark-gray boxes. The white boxes are the data sources, either internally from different pilots or externally from open and social data. The arrows between different software indicate they have direct interactions.

The MIDAS platform was developed in an iterative and collaborative way with close involvement of policy-makers and experts who informed data exploration and analysis based on their expertise. The co-created platform solves the practical policy questions proposed by the policy-makers and provides the possibility of being applied to a wider range of topics in a generally automated process. Moreover, the MIDAS platform addresses the problem of how the data can be linked, harmonized, analyzed, and visualized in a multinational framework. The scope of the specification encompasses user-interface integration, authentication and authorization, data storage, data preparation, analytics backend, visualization, and connection with external resources (Figure 1).

### Pilots

MIDAS was developed in the light of the needs of four very different pilot sites with different research topics and data sources, namely the Basque Country (Spain), Finland, Northern Ireland (United Kingdom), and Ireland. The research objectives for each pilot are listed in the table (Table 1). The MIDAS project aimed to develop a platform that could deal with a wide range of topics in the international context using machine learning models. Therefore, each pilot had a unique research topic, and separate tailored dashboards were developed for them built upon a uniform architecture.

**Table 1:** Research topics for all pilots.

**Pilot**	**Research objectives**	**Data source**
Basque	To understand what drives childhood obesity and the etiology of the childhood obesity	Controlled and open data
Finland	To understand mental health issues of young people with the support of visual analytics and analysis of available diverse datasets	Controlled and open data
Northern Ireland	To analyze the anonymized data extracted from children care system to provide new insights into a child's journey through the care system	Controlled and open data
Republic of Ireland	To study the cohort of persons with diabetes and determine the best distribution for diabetes services	Controlled and open data

### Platform Overview

As shown in Figure 1, the MIDAS Platform consists of a Policy Site Network and an External Network. The back-end analytics platform includes the Core Data Platform, tools for data harmonization (GYDRA), Analytics Backend, tools for data visualization (OpenVA), and three open and social data analytics and engagement tools in External Analytics Platforms. The policy-makers in a pilot site could adopt tools in the User Interface (UI), including the ISAACUS Metadata server, GYDRA, MIDAS Dashboard, News Media Dashboard, MEDLINE Dashboard, and Social Campaign Manager.

The MIDAS Platform is a collection of standard open-source big data processing tools, which is a modular, scalable data analytics platform along with the tools for packaging, deploying and configuring these applications in a bespoke manner.

The core services can be divided up into those which are necessary for the operation of the MIDAS Platform, and those which have been used for the development of the platform or which are optional depending on the desired usage. The required services are Hive, Spark, and HDFS; in addition, in the deployed MIDAS Platform, PostgreSQL is used for the Hive Metastore (database), but this can be changed to other database technologies. The rest of the services are deployed as part of the pilot site deployments, but these are optional services:

- OpenLDAP–used for service level authentication
- Hue–Web-UI for Hive/HDFS
- Jupyter–Web-UI for analytics notebook development
- Zeppelin–Web-UI for analytics notebook development
- PgAdmin–Web-UI for PostgreSQL
- PostgreSQL–used for Hive Metastore and/or Unified Data View data virtualization

**Figure 2:**
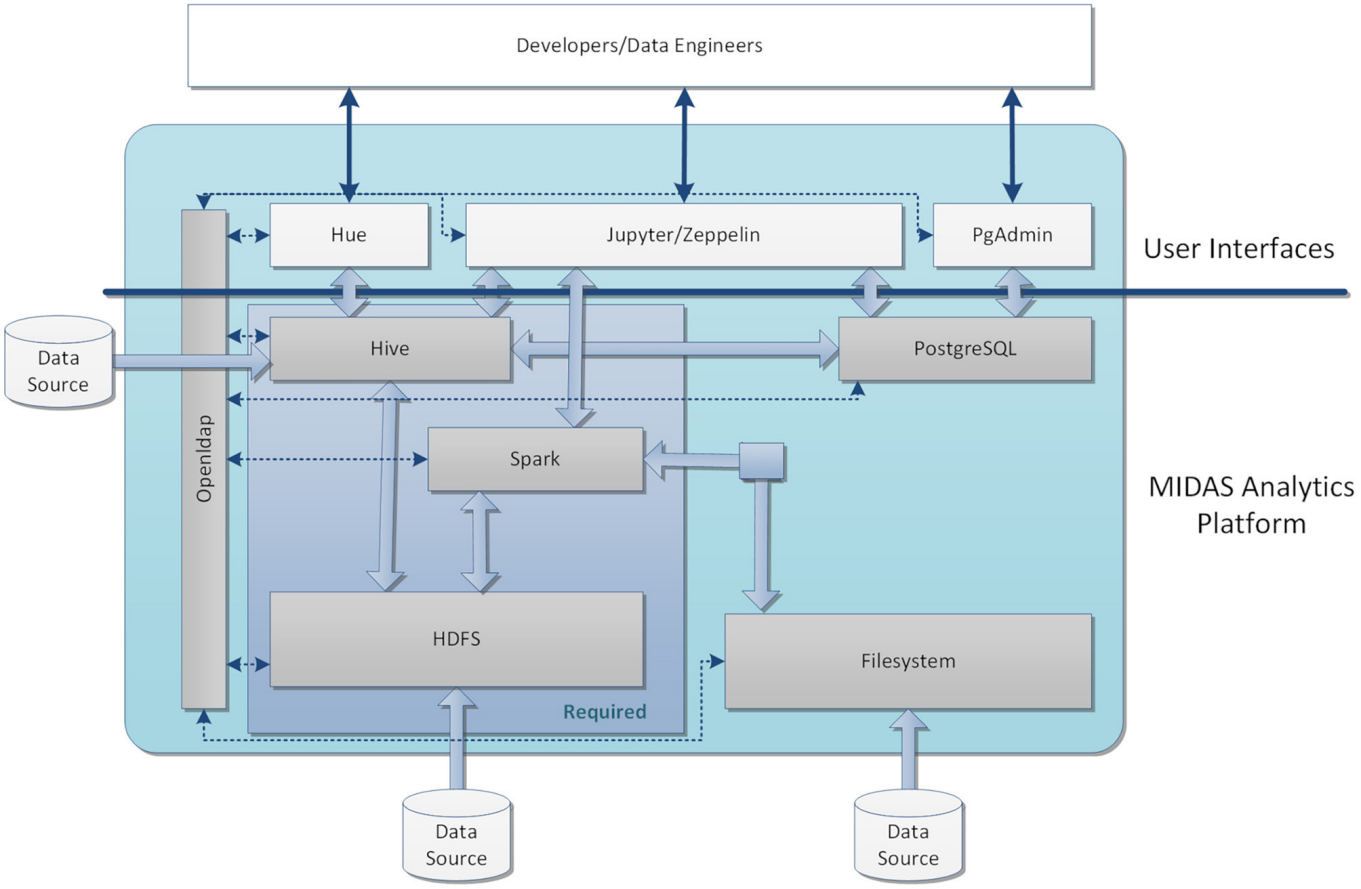
An overview of the system configured for MIDAS. The core data platform for the MIDAS stack was based on HDFS, Hive, and Spark. The data can be imported into the system through Filesystem, HDFS, or externally to Hive. HDFS was applied to store files and raw data and Hive was employed as a data warehouse for the structured data after processing. External data assets were also virtualized through the Hive interface and they could be accessed by the MIDAS tools similarly to locally loaded data assets and used within GYDRA. The UI of the analytic platform includes Jupyter Notebook with Python and PySpark for developing and testing the underlying analytics models before being implemented within the MIDAS Analytics Backend. For managing and querying the databases in Hive and PostgreSql, an open sourced interactive editor Hue was used, and Zeppelin provided support for running Spark applications. User access was managed by a local LDAP server, which provided role-based access to the user applications and underlying data stores. [2021] IEEE. Reprinted, with permission, from (31).

An overview of the Core Data Platform configured for MIDAS is given in Figure 2, including the core services of data storage and processing, user applications for interacting with these services, local user authorization and authentication, and data virtualization.

#### Data Storage and Processing

The underlying data storage for the MIDAS Platform is HDFS-based. Where data virtualization is desired this can also be provided through Hive or via PostgreSQL. Data processing engines include MapReduce, Spark and Celery for running distributed analytics workloads on the data, with Hive being employed as a data warehouse for the data within HDFS to structure it so that it can be analyzed and results provided to the MIDAS Dashboard.

#### User Applications

For development of the platform, a number of web-based applications are provided to technical users to access various services within the MIDAS Platform, including Jupyter Notebook web-application which provides entry-points to access data within HDFS/Hive and is used to develop the underlying analytics models and code before being implemented within the MIDAS Analytics Platform; Hue for working with Hive and viewing the underlying HDFS file structure; PgAdmin for interacting with PostgreSQL; and Zeppelin notebook web-application for running code on various services.

#### User Authorization and Authentication

Access to the underlying data stores and services within the MIDAS Platform is managed by a local LDAP server (running OpenLDAP), although this could be replaced with a user-specific local server or a centralized server (e.g., Active Directory) within a pilot site. This provides role-based access to the user applications as well as HDFS and Hive. Access to data within HDFS can be limited to a specific user-group or MIDAS applications, for instance, restricting access to the raw data to a pre-processing group of users or the GYDRA application.

#### Data Virtualization

Data virtualization to external data sources outside of the MIDAS platform uses Hive. This provides access to external data assets that may be held outside the MIDAS platform. External data assets will likely be existing databases (PostgreSQL, SQL Server, Oracle, etc.) which have already been preprocessed (e.g., to create a register). Once access to these external assets has been set up in Hive, they can be accessed by the MIDAS tools similarly to locally loaded data assets and used within GYDRA or pulled through to the MIDAS Dashboard.

### Data Preparation and Harmonization

The data preparation and harmonization task aimed to develop appropriate pre-processing modules for preparing the raw data to ensure that they were compatible with the agreed data representations and could be used for analysis, including for instance: data cleansing, normalization, transformation, joining, and missing value imputation. The GYDRA software (renamed from TAQIH) was developed and applied for data preprocessing and transformation (13, 14). The GYDRA is a customizable tool for facilitating the data wrangling process through interactive and visual tools, taking advantage of machine learning algorithms. The aim is to simplify the tedious and time consuming part of data analysis, allowing non-technical users to transform raw data into information ready for analysis.

**Figure 3:**
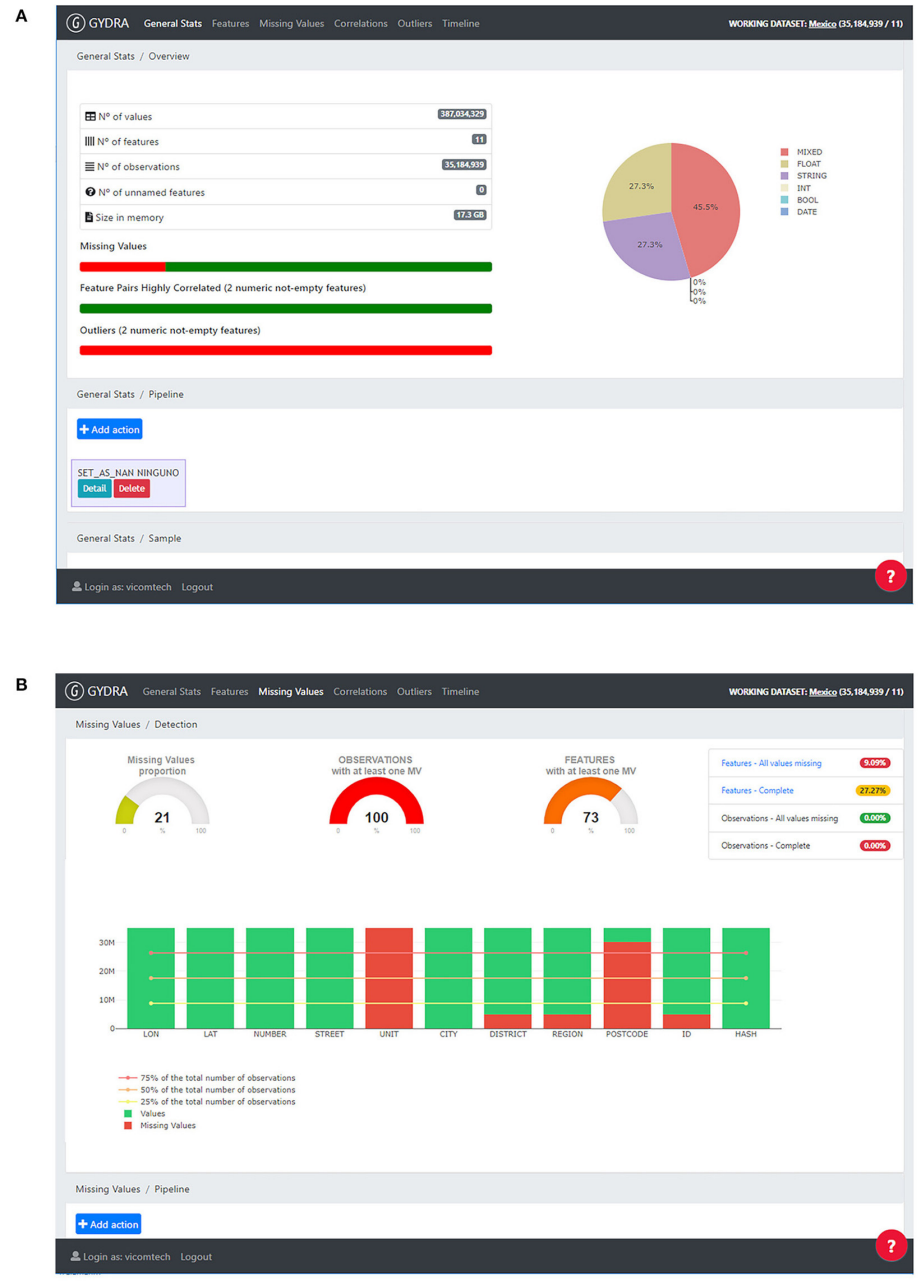
GYDRA data preparation tool UI, provided through an easy-to-use tab-based navigation approach following a common data assessment and preparation steps (i.e., General Stats, Features, Missing Values, Correlation and Outliers analysis tasks). Screenshots for two representative sections are included: **(A)** General stats–On the left side, general statistics on features and observations are provided, on the right side the variables type distribution is shown on a pie chart. On the lower part a transformations pipeline is included to add dataset transformations as their need is identified. **(B)** Missing values–On the top left area, missing value proportion is depicted for values, features and observations, on the right side indicators for complete and completely empty features and observations is provided. On the lower part, each feature is analyzed separately on bar charts representing their missing value percentage. Reprinted by permission from Springer Nature Customer Service Centre GmbH: Springer Nature, Business Information System Workshops, Chapter Enhancing the Interactive Visualization of a Data Preparation Tool from in-Memory Fitting to Big Data Sets by (14).

The GYDRA provides web interfaces to understand the content, structure and distribution of the dataset through an easy-to-use tab-based navigation approach following common data assessment and preparation steps. Figures 3A,B presents screenshots for two representative sections for general statistics and missing values, respectively. Moreover, on each tab or section of the application, a visual transformation pipeline allows the users to add a dataset transformation action after knowing the dataset's content.

As a python-centered solution, with an easy-to-use interactive UI, the GYDRA uses Celery for asynchronous distributed data-processing suitable for handling big HDFS datasets that do not fit into system memory. Additionally, through the web-based GYDRA tool, a data synchronization function allows the data owners and policy-makers to efficiently deploy prepared datasets to the analytics platform. The synchronization logic aligns the GYDRA metadata tool with the ISAACUS metadata server and updates the data warehouses further through the GYDRA backend depicted in Figure 1. The raw data of each health policy area was prepared and processed using the GYDRA tool thus making the data ready for the MIDAS Analytics Backend.

The details of data sources and data types are listed in the Supplementary Material. The technical details of the data processing section have been published (13), and another published use case can be used as an example to show how the data was processed and prepared for data analytics (15).

### Data Analytics

The MIDAS Analytics Backend provides the back-end analytics and simulation results required for the MIDAS dashboard. Apart from being a middle layer linking the data preparation and the data visualization, it supervises the user in selecting the correct data tables and data variables for chosen analytics and visualization scenarios.

The communication between the analytics and visualization layers was managed through a REST API server developed with the Flask microframework for Python. The Analytics APIs were developed to support generic exploratory data analysis (EDA), uniform across all pilot sites, as well as more specialized cross-filter dashboards and health policy simulators specific to each pilot-site.

**Figure 4:**
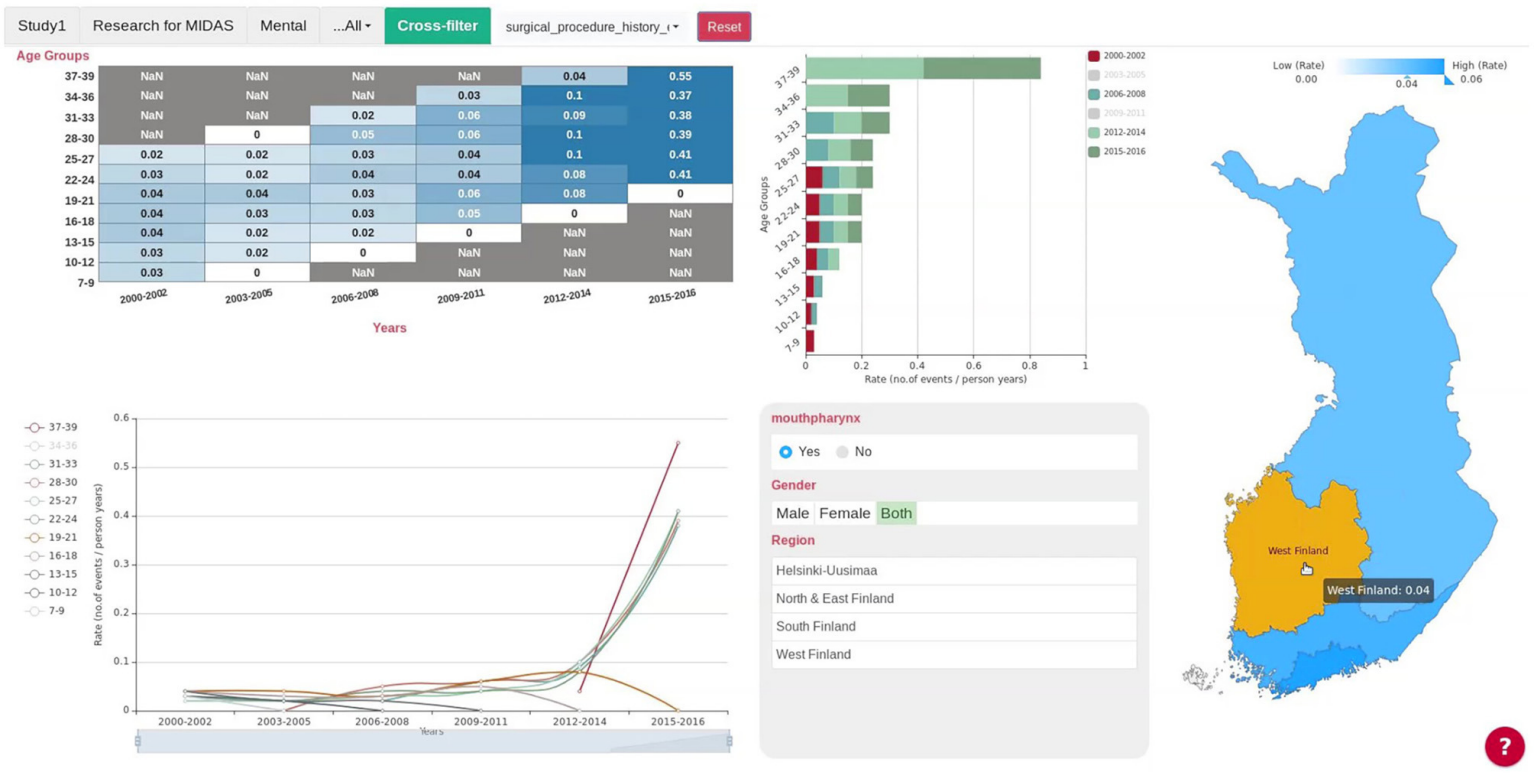
Cross-filter of the Finnish Pilot. Users can select the county or region in the map on the right and all the charts will update automatically. Gender, Region, and Age Group are the categorical variables shown in the lower panel next to the map, and users could select subgroups either by pressing the buttons on the panel or selecting the subgroups in the line chart or bar chart. The Finnish cross-filter consists of a matrix heatmap, a bar chart, a line chart and the regional map of Finland. The cross-filter of other pilots have different components.

The EDA was uniform for all pilots, providing eight types of basic visualizations for the selected variables from the harmonized data, i.e., scatter plot, heatmap, histogram, bar chart, pie chart, bubble plot and choropleth map. The cross-filter analytics for each pilot platform (Figure 4), which are interactive visualization tools (16), had the same basic principle to update their content when the user selects different values on the displayed graphs. The associated visualizations were flexible for different pilots, with the layout and categorical variables proposed by policy-makers, including components such as line chart, bar chart, and tables.

Different machine learning methods were applied for each pilot to solve their unique research questions (Table 2). Because of data protection regulations, the data-related results cannot be shown. As the main focus of the paper is on system architecture, the detailed results are not discussed and shown in this paper.

**Table 2:** Pilot-specific analytics.

**Pilot**	**Method**	**Purposes**
Basque	RandomForests/LASSO	To identify the risk factors of childhood obesity
Finland	Lexis diagram analysis	To aggregate, summarize and visualize the selected risk factors in a secure way to protect the privacy of patients
Finland	Descriptive analysis	To intuitively evaluate the health, social, and education status of the inhabitants in regional level on a yearly basis by using open data
Northern Ireland	Markov chain	To track patterns of behavior over time and to give better visualization to intuitively present how children move in and out of different types of care by estimating the probability of the transition between different types of care
Northern Ireland	LSTM Network	To predict the future status of children to improve the protection for children from the policy level
Republic of Ireland	ARIMA	To forecast the consumption of diabetic drugs

The private MIDAS GitHub repository contains branches of each pilot, consisting of API endpoints for generic EDA, cross-filter, and pilot-specific analytics. Different types of cross-filter and pilot-specific analytics were deployed on each of the pilots in an iterative process. Feedback from policy-makers on the required analytics with evaluation of results was collected in each deployment iteration, making it possible to meet the real needs of the policy-makers.

### Data Visualization

Data visualization was Deployed utilizing a three-tier architecture in the MIDAS Dashboard, including the MS Azure AD B2C authentication service (17), the OpenVA middleware framework (18), and the dedicated MIDAS UI single page application (SPA), which provides decision-making support for policy-makers with data-driven analytics from the internal and external resources.

A Single-Sign-On service was implemented between the MIDAS Dashboard and external resources through the common authentication service, mentioned above. The OpenVA framework handles the connectivity of shared SPA to local resources and dedicated external components. The MIDAS UI SPA is shared by all instances from a centralized web server and it connects to the local OpenVA instance in line with the account details of the current user. The external resources include the Social Campaign Manager, MEDLINE Publication search and News Media search dashboard.

**Figure 5:**
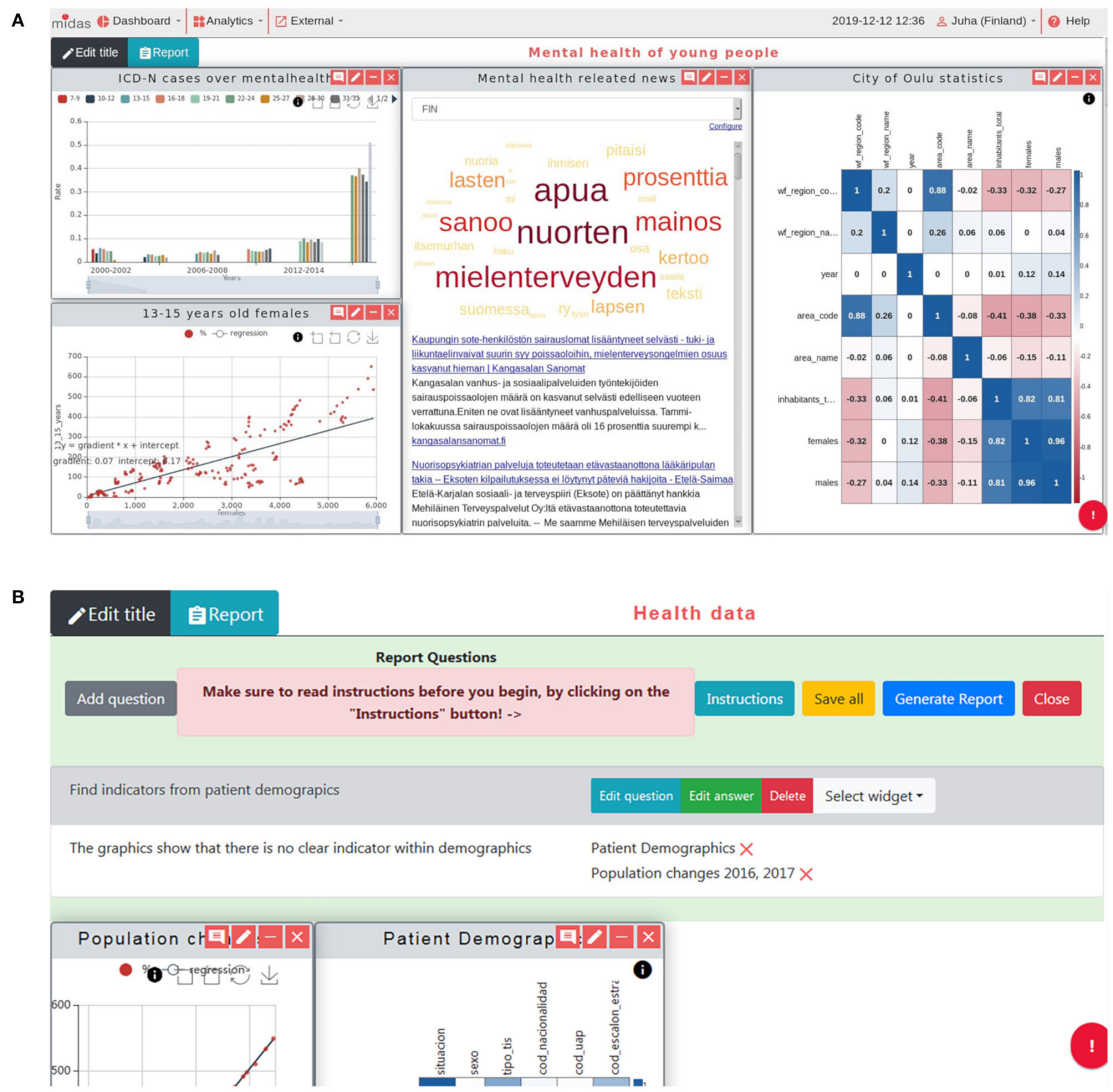
MIDAS UI screenshots. **(A)** Common view of generated dashboard. On top of the view are the menus to manage dashboards, add analytics and use the external resources. The reporting tool is located in the middle (hidden in figure) and the rest of the view is the open space for widgets. Users can freely resize and organize widgets in this space. The analytic results come from the MIDAS Analytics Backend, together with the widgets developed externally. **(B)** Reporting tool open with a single research question and an answer with two associated widgets. This tool is used to generate a PDF file, defining the research questions and answers and attaching the most suitable figures describing them from the available widgets. [2021] IEEE. Reprinted, with permission, from (31).

Through the MIDAS UI, users can generate a dashboard and interact with a widget wizard to generate the specific visualization widgets that can help them with policy decision support. Furthermore, additional pilot-specific dashboards and analytics tools were developed for each pilot, supporting each user in exploring and understanding their main research question. The MIDAS UI (Figure 5A) shows the visualized analytics results for selected datasets, together with the reporting tool illustrated in Figure 5B to allow users to generate a PDF report.

### Open and Social Data

#### Social Media Analysis

**Figure 6:**
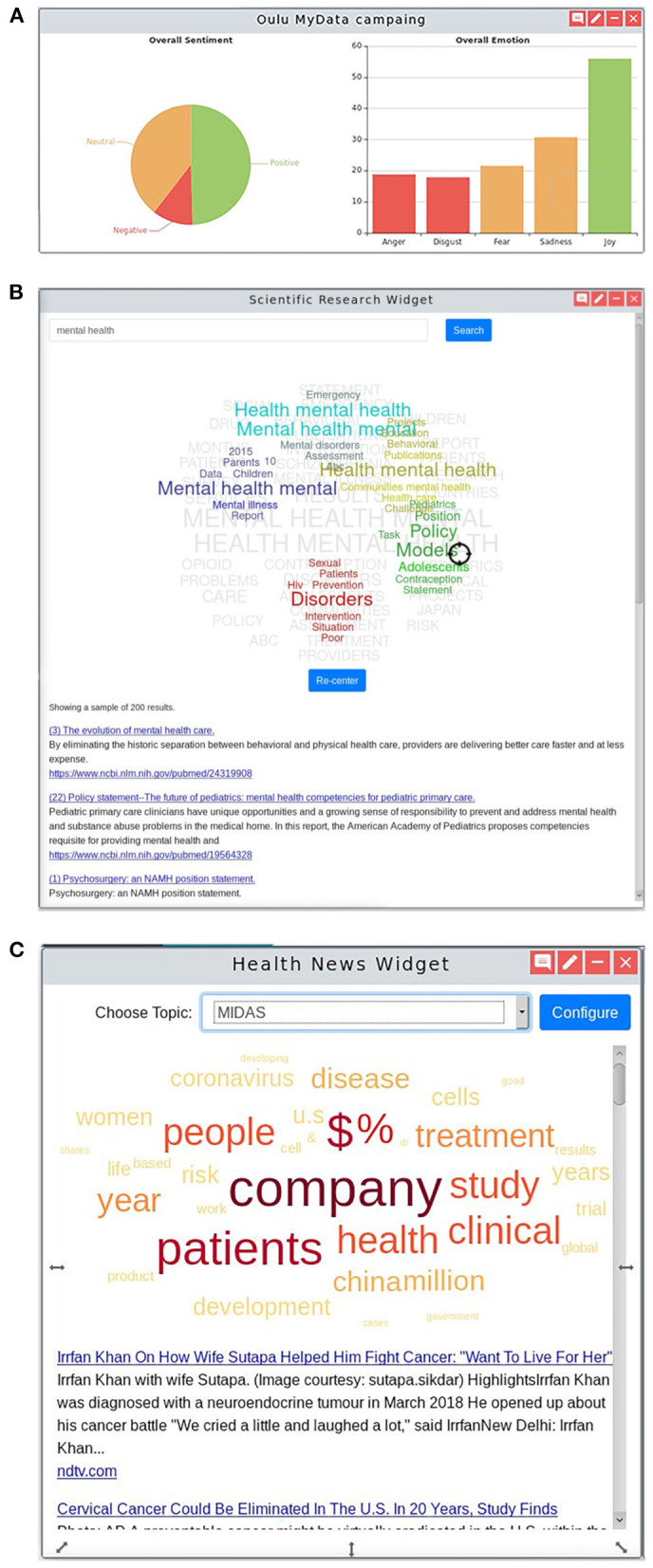
Open and Social Analytics. **(A)** Social Campaign Manager shows a high-level overview of the (i) sentiment and (ii) emotional analysis of the policy being studied by a Twitter social media campaign. The sentiment and emotions found in responses to a public online survey reaching out to the public to gather their voice on a specific health policy being considered on the dashboard. Clicking into the dashboard provides further insight including responses to particular questions and results processed using Natural Language Processing techniques showing the most common topics of conversation mentioned in the responses and the sentiment in which they were made. **(B)** MEDLINE custom widget that includes: (i) a list of the top ten MEDLINE articles with the first part of the abstract serving as a short description; (ii) a tag-cloud representing clusters of topics extracted from the MEDLINE articles including the searched keywords; and (iii) a target-shaped pointer that the user can move through the tag-cloud and by that, change the ranking of the listed articles. **(C)** News custom widget that consists of: (i) a word cloud that represents the main topics of the listed news, enabling a global perspective of the key topics before further activity; (ii) a list of news titles and first lines that are linked to the original news source; and (iii) the search choices where news are based on, defined by the filter and search options at the “Media Monitoring” menu of the external news dashboard.

Social media is considered as an important source of information for policy making, to help better understand motivations and determine public acceptance of implemented policies. The social media analysis provides insights into the public's perception and sentiments toward health policies by having members of the public engage with the created chatbots through a series of questions about a specific health policy. The questions included a number of multiple choice questions or open questions to be answered in free-form text. The free-form text responses are analyzed in real time as they are entered into the system using IBM Watson Natural Language Understanding (NLU) APIs (19), and the sentiment and emotional analysis are then displayed on the Social Campaign Manager dashboard or widget on the MIDAS UI dashboard (Figure 6A). In order to avoid bias and protect the participants' privacy, the analysis was not done on the individual level, but on the aggregated level. The bot extracted emotions from the free-form text, only giving the potential inclination of the participants. The aggregated view of these responses is the percentage of one type of emotions or opinions, which alleviated the bias generated from individual response.

The Social Campaign Manager was hosted as a microservice on the IBM Cloud platform. Twitter was used to interact with the public and the IBM Watson Assistant and Watson NLU services were used for the chatbot. The Social Campaign Manager was a separate web application for creating, running, and managing the individual campaigns. These provided the intelligence and dialog capability to interact with the user as well as performing the analysis of the conversations. The Social Campaign Manager API Server was the core application connecting these various services, providing data to the MIDAS Dashboard and the Social Campaign Manager web application. The authentication used within MIDAS platform layers is OAuth 2.0, a common industry-standard protocol for authorization.

#### MEDLINE Analytics

The MEDLINE dashboard accessible through the MIDAS platform was developed to provide dedicated text-mining tools and visualizations to enable users to extract meaningful information from the MEDLINE dataset (20, 21). The MEDLINE dataset was indexed using the ElasticSearch, and visualized through an open source tool Kibana (22). The purpose of the dashboard was to provide users with tools to explore the insights of published biomedical research, in an intuitive manner. The main advantage is the dynamic article prioritization (ranking). The user enters a few keywords in the search box and results are shown (Figure 6B). This visual interactive widget helps surface information that one is looking for by re-ranking the top 10 articles, letting the users interact with the index of the results, getting them closer to the scientific information that they are looking for.

Each topic dashboard was developed through extensive interactions with the pilot sites, improving the understanding of how the tools could be used to address specific use cases. The MEDLINE knowledge was also served directly at the MIDAS platform by a widget that also allowed for Lucene queries and for the user to interact with a pointer over a tag cloud of related topics to alter the order of scientific articles provided as result of the query.

#### News Media Analysis

MIDAS provides users with tools to monitor specific health topics in the worldwide and local news. The news media analysis tool is available through the platform (Figure 6C), enabling the monitoring of worldwide news outlets and the enriching of these news articles with data from the MEDLINE knowledge base (23). Each pilot region in the MIDAS project has its own live news source which can be accessed via the dedicated news data exploration dashboard served by the Event Registry news engine and through a widget within the MIDAS Dashboard UI (24). In addition to setting up the pilot-specific data streams, the underlying data sources for Event Registry were improved to better support Finnish and Basque language news coverage, adding to the 60+ languages available. In addition to the news media tools, a MeSH Classifier tool was developed which enables classification of news articles (and any text snippets) with MeSH terms. The system is available through a web portal and a REST API, and includes a NodeJS wrapper for direct inclusion into other systems (25).

### Implementation

Given the heterogeneous nature of the various data sources, policy environments and stakeholder perspectives, the platform development followed an agile, user- centerd design approach to ensure that user needs were met across the consortium and beyond. User-centered design approach included a co-design workshop, an iterative platform evaluation, and feedback integration. The co-design workshop was attended by approximately 80 participants, including a mixture of consortium members and external stakeholders. The professional backgrounds of attendees were diverse and included policy-makers, civil servants, academic experts, and industry representatives. The workshop took participants through a staged process, which included the development of “personas” (i.e., typical users of the system), the identification of “user stories” (simple, non-technical descriptions of user requirements), and the brainstorming of “wireframes” (interface design ideas) on paper and online. The results from the workshop were subsequently collated, analyzed and distributed among consortium partners to inform the future development of the MIDAS platform (26).

We conducted three rounds of user experience testing to help improve the intermediate prototypes, methodology and results of the initial round are reported in (27). A combination of heuristic and formative user-centered evaluation methods was employed, providing feedback from both usability experts and evaluating prototypes with real users. A rigorous test protocol was jointly developed by consortium members, led by usability testers from Ulster University's UX Lab. The usability testing protocol was informed by Ulster's UX-Lab having carried out a range of usability tests on medical devices, software and data visualizations (28, 29). The participants included data analysts and policy makers, a more detailed demographic statistics can be seen in Supplementary Table 2.1 in the Supplementary Material. We guided the participants to finish a list of tasks and collected their feedback and suggestions for further improvements. The user experience testing helped successfully identify the potential problems, and improvements were achieved after incorporating user feedback.

## Results

The developed MIDAS platform consists of several dashboards, including Exploratory Data Analysis (EDA) (Figure 5A), cross-filter dashboard (Figure 4), pilot-specific analytics dashboard, and social media dashboards (Figure 6).

The final versions of the pilot platforms were evaluated by policy makers from all pilots based on the Key Performance Indicators (KPI) (Supplementary Table 2.2 in the Supplementary Material). The second column is the demands proposed by policy makers, and the third column is the corresponding function on the MIDAS platform. All KPIs were successfully achieved and the platform has received positive feedback from stakeholders on its capacity to integrate and analyze previously fractured heterogeneous data. Furthermore, the ability to produce new knowledge and results that are actionable by health policy-makers was demonstrated. The custom-tailored analytics solved the practical questions for the health policy-makers and gave them insights for possible future interventions. The platform can be easily manipulated by users without technical background by following the User Guide (30).

## Discussion

### Principal Results

The core user groups of these tools are mainly business users, dashboard users and in-house analytics teams. In contrast, the MIDAS platform was co-created by academia, industry, and crucially, healthcare staff, health policy-makers, patients and citizens thus ensuring the solution's design and development has been user-led. With this user-centered approach, the MIDAS platform guides its users through all steps of the data analytics pipelines. Besides, data blending is restricted according to the prior knowledge of the original data in the data processing procedure. These restrictions assist the user in selecting only suitable variables for a chosen visualization, thus producing meaningful analytics and visualization results.

Because of the flexibility of the open data tools, they can be quickly adjusted to study the most urgent topics, as a result, MIDAS recently presented a fast response to the COVID-19 global initiative (31). This impactful public health event was addressed through the worldwide news, offering the customized news streams through the MIDAS news widget, to help the pilot site use cases to better track news and relate it to their own priorities.

In order to maximize the sustainability of the MIDAS platform beyond the lifetime of the project, we explored a range of mechanisms for coordinating further development and marketing activities among the project contributors post-project. After detailed partner and stakeholder engagement we determined that the establishment of a MIDAS Open Source Foundation would be the most suitable approach. New regions, cities, and organizations from Scotland, France, and Spain have confirmed their interest, with more public sector policy departments noticing the platform capability of addressing similar problems in their area in future.

### Comparison With Prior Work

The MIDAS platform tries to maintain the privacy of each stakeholder by keeping their sensitive health data in-house. Other commercial tools like Tableau, PowerBI, or QlikView often require a connection to external services, while all layers of the MIDAS platform are hosted inside the stakeholder's trusted zone. Moreover, they are general purpose solutions that do not consider the specific challenges of public health data, nor the user stories of the target MIDAS audiences. Therefore, each layer of the MIDAS platform supports a secure data analytics pipeline and minimizes data-leakage. Additionally, the learning curve of some commercial tools can be steep, requiring specialized training. In terms of advanced analytics capabilities, Tableau provides some advanced analytics features but with external integration, PowerBI has core competency and integration, while QlikView does not offer any advanced analytics features.

### Conclusions

This study has demonstrated the value of a secure, effective and integrated solution that deals with health data to harness the potential of underutilized healthcare data and provide support for health policy decision making. The MIDAS platform was successfully implemented within the four pilot regions and has received positive feedback from stakeholders on its capacity to turn large amounts of previously isolated data into actionable information to inform health policy making and risk stratification through the applications and visualizations of advanced analytics. By delivering the MIDAS platform as an innovative and state-of-the-art solution, we have successfully provided a tool with fully functioning architecture that can potentially transform the way health policies are developed, evaluated and implemented, which will ultimately enable impactful improvements in public health and the quality of life amongst European citizens and beyond. Besides, the platform has successfully demonstrated that it is transferable, sustainable and scalable across policies, data and regions.

## Data Availability Statement

The data that support the findings of this study are available from MIDAS but restrictions apply to the availability of these data, which were used under license for the current study, and so are not publicly available.

## Author Contributions

XS, GN, SF, MB, DR, GE, JPC, JuP, PP, and JW: analyzed the data, contributed reagents, materials, analysis tools, and wrote the paper. All authors were involved to conceive and design the study, reviewed and interpreted the results, commented on manuscript, contributed to revision, and read and approved the final version.

## Funding

This work was supported by KU Leuven: Research Fund (projects C16/15/059, C3/19/053, C32/16/013, and C24/18/022), Industrial Research Fund (Fellowship 13-0260), and several Leuven Research and Development bilateral industrial projects, Flemish Government Agencies: FWO [EOS Project no 30468160 (SeLMA), SBO project S005319N, Infrastructure project I013218N, TBM Project T001919N; Ph.D. Grants (SB/1SA1319N, SB/1S93918, SB/151622)]. This research received funding from the Flemish Government (AI Research Program). BD and XS are affiliated to Leuven.AI–KU Leuven institute for AI, B-3000, Leuven, Belgium. VLAIO [City of Things (COT.2018.018), Ph.D. grants: Baekeland (HBC.20192204) and Innovation mandate (HBC.2019.2209), Industrial Projects (HBC.2018.0405)], European Commission: This project has received funding from the European Research Council (ERC) under the European Union's Horizon 2020 research and innovation programme (Grant Agreement No 885682), (EU H2020-SC1-2016-2017 Grant Agreement No.727721: MIDAS), and KOTK foundation.

## Conflict of Interest

SF was employed by Analytics Engines. JPC, MG, and LS were employed by Quintelligence. PP was employed by International Business Machines Corporation. The remaining authors declare that the research was conducted in the absence of any commercial or financial relationships that could be construed as a potential conflict of interest.

## Publisher's Note

All claims expressed in this article are solely those of the authors and do not necessarily represent those of their affiliated organizations, or those of the publisher, the editors and the reviewers. Any product that may be evaluated in this article, or claim that may be made by its manufacturer, is not guaranteed or endorsed by the publisher.
