# Supplementary material for: System Architecture of a European Platform for Health Policy Decision Making: MIDAS
Source: Front Public Health. 2022 Mar 31;10:838438. doi: 10.3389/fpubh.2022.838438 (PMC9008448; doi:10.3389/fpubh.2022.838438)
Supplement: Supplementary file 1 [file Data_Sheet_1.docx]

Supplementary Material

# Supplementary Data

## Basque Country

Osakidetza is the basque public health provider which depends on the Department of Health of the Basque Government. Currently Osakidetza provides service to more than 2.200.000 patients, through 16 hospitals and more than 300 primary health centres. Osakidetza datasets (e.g. prescriptions, imaging data, information systems) both for primary care and hospital are integrated and run under a unique electronic health record (EHR). Osakidetza is organized in different areas called OSI (Organización Sanitaria Integrada – Integrated Healthcare Organization) that cover a region of the Basque Country.

This dataset is composed of sub datasets extracted from primary care, specialty care and hospitals databases that contain different health information about the patient for a global view of his/her health. The information about the child that this dataset gathers is information about the patient, diagnosis, medical forms, medical appointments and prescription information. In addition to this information about the child, the dataset collects information from the primary and specialised care forms of the child’s mother. This dataset collects information about 800.000 children, starting from those under 18 years on year 2000.

Open Data Euskadi is an initiative framed within the Basque Country’s Open Government policy. Basque Government publishes public data (those that have not been restricted due to privacy, security or intellectual property reasons) through the Open Data Euskadi platform.

Examples of potential variables for an initial analysis for government open data include: population density, urban density, temperature, green space, traffic density, bicycle lane density etc.

## Finland

The main data sources for the Finnish pilot are the city of Oulu database, THL national databases and Northern Finland Birth Cohorts (NFBC) databases.

THL data is collected nationally from health care providers like hospitals and cities. The data is updated annually and is integrated based on the personal ID. Examples of the THL data include hospital register and population-study-based data mapped over two different geographical distributions: Finnish large regions and Constituencies.

Another significant data source from Finland is the Northern Finland Birth Cohorts. They comprise collections of data and biological samples from large population studies that are administered by the Northern Finland Birth Cohorts’ Project Center at the Medical Faculty, University of Oulu. The final number of subjects in the cohort is over 20 000. The original data has been supplemented by data collected with postal questionnaires at the ages of 7, 8 and 15 / 16 years with various hospital records and statistical register data. The cohort data includes partially the same variables as THL data.

The City of Oulu dataset (OUKA) gathers information about more than 200 000 inhabitants of the city of Oulu. This data source contains various types of general population data about inhabitants living in different city areas. The city level data fosters city level policy creation and decision making. It contains details from Health and welfare, Social and Family Services, and Education and Culture. The actual data preprocessed from Paavo open data lake of Statistics of Finland. The data variables are selected in relation to Oulu regional policy targets from open data source and it covers the whole population of the city between years 2012 to 2017.

## Northern Ireland

The project analyses data from Health and Social Care (HSC) in Northern Ireland for patients/clients in the age range 0-25 during the time period 1st January 2004 to 31st December 2014. It may be possible to add other datasets such as Child Health registrations, inoculation/vaccination records, Dental Registration. The data shall be used to examine the health outcomes of policy decisions made in the past (e.g. around Looked After Children) and to model the likely impact of new policies.

For the Government Open data Northern Ireland Statistics and Research Agency (NISRA) portal has been used, which is available at <https://www.nisra.gov.uk/>. NISRA is the principal source of official statistics and social research on Northern Ireland. NISRA conducts the Census of Population every 10 years which every household in Northern Ireland must complete by law. It is used by central and local government, health authorities and other organisations to plan and provide future services.

## Republic of Ireland

Datasets are encrypted as appropriate and parties bound by HSE Service Provider Confidentiality Agreement where appropriate. Sources include: HSE Hospital In-Patient Enquiry (HIPE) and HSE Primary Care Reimbursement Service (PCRS) with a focus on hospital admissions, discharges and prescribing datasets.

Additionally, to support the analysis, the government generated datasets containing pharmacies list, prescription drugs information, hospitals and population per county were added. In the Republic of Ireland, the counties are, in general, the basis for local government, planning and community development purposes, are governed by county councils and are still generally respected for other purposes.

Open datasets are available through Open Data Portal accessible at <https://data.gov.ie>. Health-related specific open data is available at <https://data.gov.ie/data/search?theme-primary=Health>. This data refers to hospitals in the Republic of Ireland aggregated by single hospitals and population information per Irish county aggregated by Irish counties.

Table 1.1 An overview of available open and controlled datasets for all pilots

| Pilot | Data Name | Data Source | Sample size | Availability |
| --- | --- | --- | --- | --- |
| Basque | Child and Mother Patient care data | Osakidetza - Basque public health care provider | 800,000 children since 2000 | Controlled |
| Basque | Air / Water quality, National statistics, City planning | Open Data Euskadi (Basque Government open data)  (http://opendata.euskadi.eus/) | Not applicable | Open |
| Finland | NFBC (Northern Finland Birth Cohort) | University of Oulu  (<https://www.oulu.fi/nfbc/>) | Over 20,000 individuals | Controlled |
| Finland | Patient Health and social care data | the Finnish Institute for Health and Welfare (THL) | Cohort at age of 7-24 in 2000 | Controlled |
| Finland | Key statistical indicators per area | Statistics Finland - Paavo  (<https://www.stat.fi/tup/paavo/index_en.html>) | About 200,000 inhabitants | Open |
| Northern Ireland | Child health and social care data | Health and Social Care  Northern Ireland | About 100,000 children from 2004 to 2018 | Controlled |
| Northern Ireland | Northern Ireland Multiple Deprivation Measure 2017 | Northern Ireland Statistics and Research Agency | A ranking of 890 Super Output Areas | Open |
| Ireland | Hospital In-Patient Enquiry (HIPE)  Primary Care Reimbursement Service (PCRS) | Health Service Executive | HIPE: 195,918 records  PCRS:138,713,639 records | Controlled |
| Ireland | Census and List of Hospitals in Ireland | Ireland's Open Data Portal  (<https://data.gov.ie/>) | Census: population (2011)  Hospitals: 57 hospitals | Controlled |

# Supplementary Tables

Table 2.1 The background information of the participants of user experience testing

|  | Version 1.0 | Version 2.0 | Version 2.1 | Version 3.0 |
| --- | --- | --- | --- | --- |
|  |  |  |  |  |
| N | 12 | 5 | 7 | 3 |
| Age | / | 47.2 | 51.9 | 52 |
| Gender |  |  |  |  |
| Female | 7 | 2 | 5 | 1 |
| Male | 5 | 3 | 2 | 2 |
| Occupation |  |  |  |  |
| Data Analyst | 7 | 5 | 4 | 2 |
| Policy Maker | 5 | 0 | 3 | 1 |

Table 2.2 Key Performance Indicators

| Pilot | Updated KPI | MIDAS Platform data and components to meet the KPIs |
| --- | --- | --- |
| Basque | KPI 1: To provide and clearly identify both crude and adjusted rates.  KPI 2: To provide information at different granularity levels, adequate for each stakeholder.  KPI 3: To cover time, place, and person analysis for epidemiological studies | KPI 1: These were calculated by cross-filtering tool as new diabetes cases adjusted rates per age, sex, region, area & registration year.  KPI 2: Basque cross-filtering tool user interface was developed to answer this. OSI (trust) and UAP (primary care unit) level.  KPI 3: Also this is covered by Basque cross-filtering tool with gender and age filters (person dimension) over different years (time dimension) and for two different granularity level health area distributions (place dimension) |
| Finland | *National level:*  KPI 1: Support understanding and managing the challenges of mental health issues with young people.  KPI 2: Enable analysis of the available datasets to identify the leverage points in effective preventative measures.  KPI 3: Enable the use of visual analytics to assess the impact of different factors and indicators for policy decisions.    *Regional level (City of Oulu):*  KPI 4: Enable city data and open data evaluations from various sources in social and healthcare units in the stakeholder organizations to support population and area comparisons for preventive policy making. | *National level:*  KPI 1: Main contribution is given by the cross-filtering tool for Lexis analysis results.  KPI 2: The system dynamic model developed for the national level research question and procedure to create the model  KPI 3: Lexis analytics related single Lexis result visualizations on Dashboard and simulator interface of system dynamic model    *Regional level (City of Oulu):*  KPI 4: City of Oulu level open data was collected to answer this question. The data is visualized with cross-filtering tool and single data visualizations options on MIDAS Dashboard. The young people's personal opinions from the Northern Finnish regions were covered by the NFBC dataset. |
| Northern Ireland | KPI 1: Analysis of the available datasets to identify effective preventative measures  KPI 2: A longitudinal analysis and track a cohort of Looked After Children as they move in and out of care, use a variety of health services, and look at patterns of behaviors and changes over time.  KPI 3: The use of visual analytics to assess the impact of changing variables and indicators on policy decisions  KPI 4: Harmonization and integration of multiple data sources | KPI 1: Done within data integration work. Data analytics include LSTM simulator that allows policymakers to identify children at high risk. Analytics for Department of Health (DoH) allows exploring different subgroups of children per selected categories.  KPI 2: Answered by pilot-specific analytics and related visualizations on MIDAS Dashboard: Markov Chain analysis, LSTM simulator and DoH analysis  KPI 3: Visualization of KPI2 results  KPI 4: Done as a part of data integration work |
| Republic of Ireland | KPI 1: The provision of an adoptable intelligent analytics platform for stakeholder use  KPI 2: Identification of the cohort of persons with diabetes in the Republic of Ireland  KPI 3: Determining information shared to facilitate better use of resources and services for diabetic people  KPI 4: Determining the best fit for diabetes services and their locations based on population based geographical needs  KPI 5: Identification of improved outcomes if certain ‘data insight’ based policies are implemented | KPI 1: MIDAS Platform integration on pilot environment  KPI 2: Dedicated tables and variables to identify these cohorts were created for the database  KPI 3: The cross-filtering tool was developed to answer the (diabetes) drug usage questions  KPI 4: Partially answered by the cross-filter tool geographical representation of drug usage  KPI 5: This KPI was found not possible to answer as related data is not available for the project |
